# Supplementary material for: Body height and waist circumference of young Swiss men as assessed by 3D laser-based photonic scans and by manual anthropometric measurements
Source: PeerJ. 2019 Dec 12;7:e8095. doi: 10.7717/peerj.8095 (PMC6931388; doi:10.7717/peerj.8095)
Supplement: Table S1 [file peerj-07-8095-s001.docx]

**Supplementary Table 1**

| **Visceral fat** |  |  |  |
| --- | --- | --- | --- |
| **Manual WC** | **Coeff** | **95% CI** | **p** |
| <94.0 | ref |  |  |
| 94.0-101.9 | 1.94 | 1.53 - 2.34 | <0.001 |
| >102.0 | 4.57 | 3.7 - 5.44 | <0.001 |
| **Automatic scan WC** |  |  |  |
| <94.0 | ref |  |  |
| 94.0-101.9 | 1.85 | 1.34 - 2.36 | <0.001 |
| >102.0 | 3.43 | 2.73 - 4.13 | <0.001 |
| **Adjusted scan WC** |  |  |  |
| <94.0 | ref |  |  |
| 94.0-101.9 | 1.61 | 1.14 - 2.07 | <0.001 |
| >102.0 | 3.19 | 2.42 - 3.96 | <0.001 |
| **Relative fat mass** | |  |  |
| **Manual BMI** |  |  |  |
| <18.5 | -6.63 | -13.77 - 0.52 | 0.068 |
| 18.5-24.9 | ref |  |  |
| 25.0-29.9 | 11.28 | 8.21 - 14.34 | <0.001 |
| >=30 | 20.45 | 14.54 - 26.37 | <0.001 |
| **Standard scan BMI** |  |  |  |
| <18.5 | -6.63 | -13.77 - 0.52 | 0.068 |
| 18.5-24.9 | ref |  |  |
| 25.0-29.9 | 11.28 | 8.21 - 14.34 | <0.001 |
| >=30 | 20.45 | 14.54 - 26.37 | <0.001 |
| **Straight scan BMI** |  |  |  |
| <18.5 | -6.59 | -13.81 - 0.64 | 0.073 |
| 18.5-24.9 | ref |  |  |
| 25.0-29.9 | 11.31 | 8.20 - 14.43 | <0.001 |
| >30 | 20.49 | 14.50 - 26.48 | <0.001 |
