# Supplementary figures and images for: Body height and waist circumference of young Swiss men as assessed by 3D laser-based photonic scans and by manual anthropometric measurements
Source: PeerJ. 2019 Dec 12;7:e8095. doi: 10.7717/peerj.8095 (PMC6931388; doi:10.7717/peerj.8095)

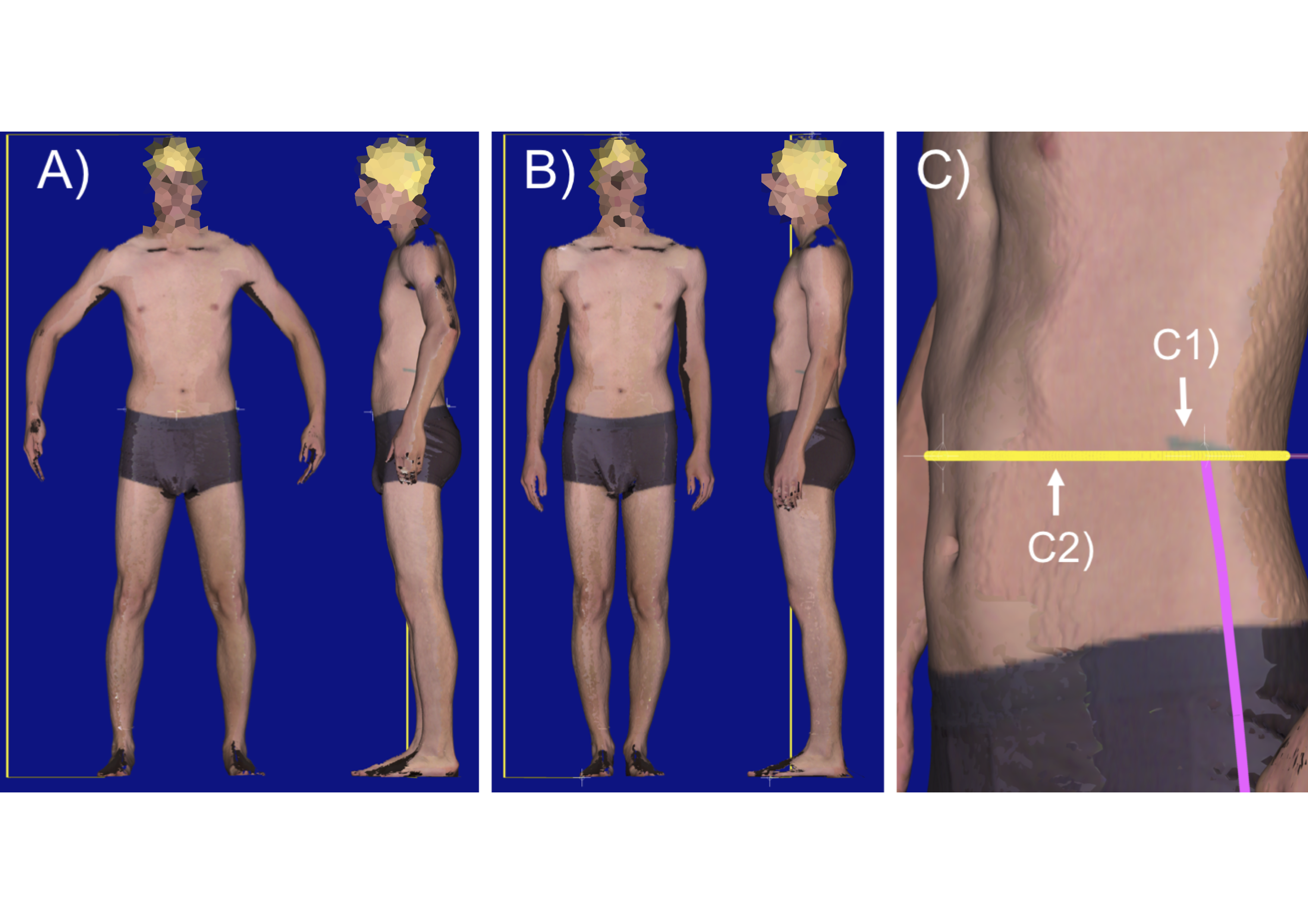

Supplement: Figure S1 — (A) standardized position specified by the scanner manufacturer: standing in an upright position, both feet positioned on marks on the scanner platform (spaced approximately 30 cm apart), arms slightly bent in the ellbow and held slightly separated from the body, head in accordance to the Frankfurt Horizontal Plane. (B) standing straight in an upright position with feet together (same position as during the manual height measurements with the stadiometer). (C) the measurement line of the software was manually moved to match the pen mark of the manual WHO measurement point on the scans. [file peerj-07-8095-s003.png]

WC

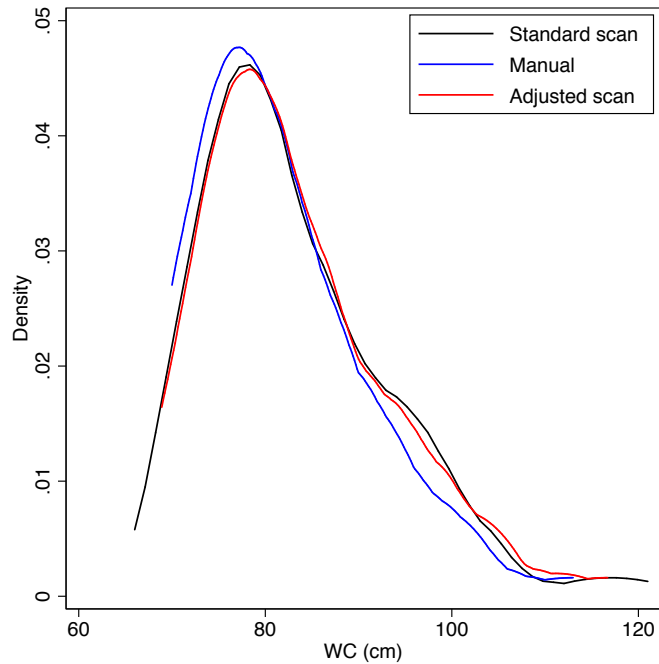

Height

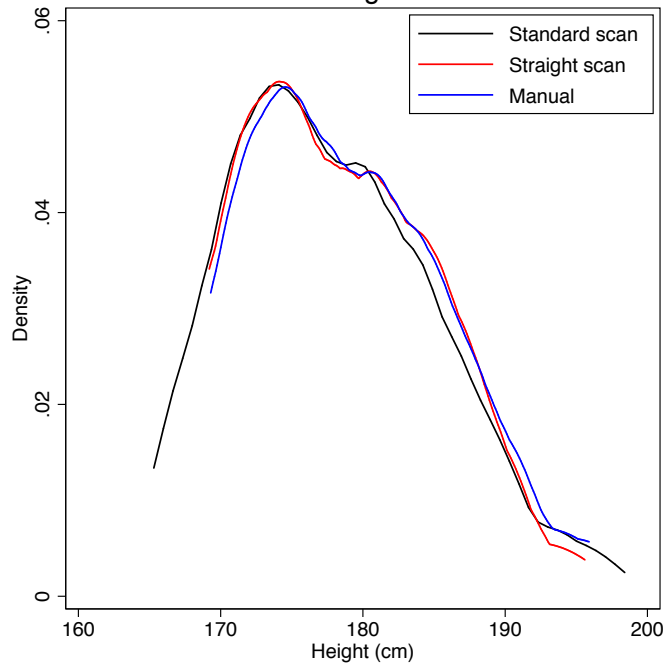

BMI

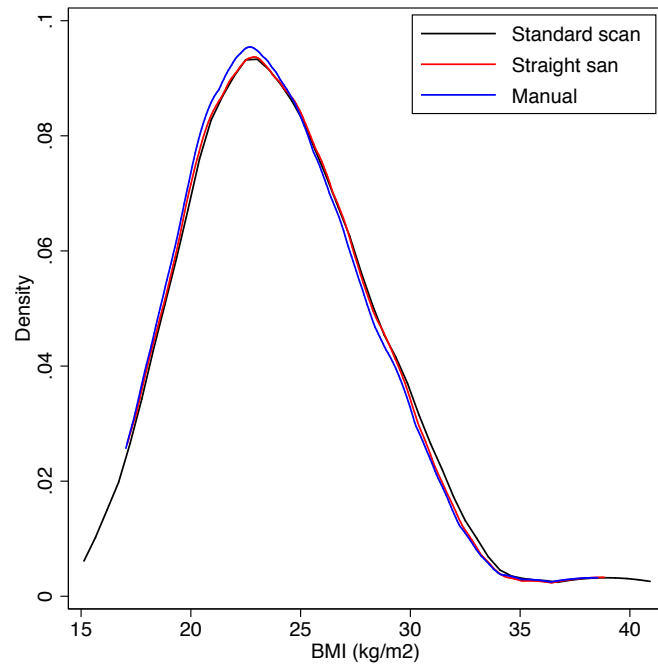

Supplement: Figure S2 — The distributions for WC, height, and BMI were not entirely symmetrical, neither for manual measurements, nor for the two scanner positions. [file peerj-07-8095-s004.pdf]
